# Isolation and characterization of a duck-origin goose astrovirus in China

**DOI:** 10.1080/22221751.2020.1765704

**Published:** 2020-06-02

**Authors:** Feng Wei, Jing Yang, Yueming Wang, Hao Chen, Youxiang Diao, Yi Tang

**Affiliations:** a College of Animal Science and Technology, Shandong Agricultural University, Tai’an, People’s Republic of China; b Shandong Provincial Key Laboratory of Animal Biotechnology and Disease Control and Prevention, Tai’an, People’s Republic of China; c Shandong Provincial Engineering Technology Research Center of Animal Disease Control and Prevention, Tai’an, People’s Republic of China; d College of Life Sciences, Qufu Normal University, Qufu, People’s Republic of China

**Keywords:** Novel goose astrovirus, ducklings, next-generation sequencing, phylogenetic analysis

## Abstract

In 2019, a new type of infectious disease characterized with haemorrhage and swellings of kidneys, occurred on commercial duck farms in Shandong province, China. Our systematic investigation led to the isolation of an astrovirus, designated AstV-SDTA strain and was isolated from a diseased duckling using LMH cells. Similar clinical symptoms were reproduced by experimental infection using the AstV-SDTA strain. The complete genome sequencing characterization of AstV-SDTA was conducted using next-generation sequencing (NGS) technique on Illumina HiSeq platform, and used polymerase chain reaction method to verify the NGS results for the obtained whole sequences. Phylogenetic analysis revealed that AstV-SDTA strain belongs to a novel goose astrovirus (GoAstV) branch of avian astroviruses, and the nucleotide homology based on the complete genome sequences among AstV-SDTA and other GoAstV strains deposited in Genbank was 97.2–98.8%. Taken together, these results suggest that the cross-species transmission of novel GoAstV between domestic waterfowl is possible. Further surveillance of novel GoAstV in poultry are needed in order to gain a better understanding of both the molecular and evolutionary characteristics of novel GoAstV.

## Introduction

Astroviruses (AstVs) are a single-stranded, positive-sense RNA viruses with a genome of approximately 6.8 to over 7.9 kb [1], consisting of a 5′-untranslated region (UTR), three open reading frames(ORFs), a 3′UTR and a poly (A) tail [2]. Currently, Astroviruses are classified into two genera: namely Mamastroviruses (MAstVs) and Avastroviruses (AAstVs) according to the different types of infected hosts [3]. In 1975, astrovirus was first identified in faeces of infants [4]. At present, AstVs infections have been found to infect a variety of animal species, including: cattle, sheep, pigs, chickens, turkeys, ducks, dogs, cats, and rat [5–9]. Thus far, AstVs have been reported as showing associations with duck hepatitis, which is historically known as duck hepatitis virus type 2 (DHV-2) and duck hepatitis virus type 3 (DHV-3). Originally classified as a picornaviruse and later reclassified as astroviruses based on virion morphology and/or subsequent comparison of RNA-dependent RNA polymerase (RdRp) sequences [10–12]. Since 2016, a novel disease characterized by gout, haemorrhage and swellings of kidneys has affected the goslings in a number of provinces of China, which has caused serious economic losses are between 1.2 and 1.5 billion yuan [13–15].

In 2019, an epidemiological investigation was conducted in commercial duck farms. A disease characterized by haemorrhage and swellings of kidneys was detected, and very rare affected ducklings showed urate deposition in the internal organs and joint cavity. In the present study, a systematic investigation of the pathogens causing the disease was carried out, which covered epidemiology, pathogen isolation and identification tests, reproduction experiments and genomic sequencing. The results showed that the causative agent of the newly emerging duckling disease in China is caused by a novel goose astrovirus (GoAstV) strain.

## Materials and methods

### Bacterial culture and viral nucleic acids detection

For bacteriological diagnosis, fresh tissue samples (e.g. liver and kidney) were collected and then inoculated onto tryptic soy agar plates (BD Science, MD, USA) containing 2% foetal bovine serum (FBS; Hyclone, Beijing, China), and were incubated at 37°C with 5% CO_2_ for 48 h. The spleen, liver, and kidney tissue from different duck flocks were pooled for the detection of fowl adenovirus [16], duck plague virus [17], duck Tembusu virus [18], duck astrovirus [19], duck reovirus [16], duck circovirus [20], goose haemorrhagic poly-omavirus [21] and goose parvovirus [22], respectively (Table 1).

**Table 1.** Primers used in this study for detection of the viruses.

Primer name	Sequence (5′→3′)	gene	Product size (bp)
FAdV F	CAACTACATCGGGTTCAGGGATAACTTC	Hexon gene of fowl adenovirus	766bp
FAdV R	CCAGTTTCTGTGGTGGTTGAAGGGGTT		
DTMUV F	GCCACGGAATTAGCGGTTGT	E gene of duck Tembusu virus	401bp
DTMUV R	TAATCCTCCATCTCAGCGGTGTAG		
GHPV F	GAGGTTGTTGGAGTGACCACAATG	VP1 gene of goose haemorrhagic polyomavirus	144bp
GHPV R	ACAACCCTGCAATTCCAAGGGTTC		
GPVF	AGACTTATCAACAACCATCAT(C) T	VP1 gene of goose parvovirus	779bp
GPVR	TCACTTATTCCTGCTGTAG		
DRVF	CTTTTTGAGTCCTTGTGCAGCCATG	NS gene of duck reovirus	1065bp
DRVR	GTAAGAGTCCAAGTCGTGGCAGAGG		
DAstVF	CAGGGAGTGGTTGTTGCAGAT	ORF1a gene of duck astrovirus	482bp
DAstVR	TTCTCACTT TGTTCTCGCGG		
DucVF	CTCGAGTGAACCCGGTGAACTGACC	Cap gene of duck circovirus	753bp
DucVR	GAATTCATGCGACGCAGCACCTATC		
DPVF	CGGAATTCCAAAACGCCGCACAGATGAC	gC gene of duck plague virus	432bp
DPVR	CCCTCGAGGTATTCAAATAATATTGTCTGC		

### Sample collection

Eighteen affected ducklings and sixty cloacal swab samples were collected from each of the 4 different commercial duck farms (i.e. A in Taian County, B in Liaocheng County, C in Xuzhou County, and D in Zhengzhou County) in Shandong, Jiangsu, and Henan provinces of China, from July to October 2019. These collected samples were tested by reverse transcriptase-polymerase chain reaction (RT–PCR) and quantitative reverse transcriptase-polymerase chain reaction (qRT-PCR) assay for the presence of novel GoAstV viral RNA as described previously [23]. Meanwhile, the liver and kidney samples of the diseased ducklings from different commercial duck farms were collected for virus isolation.

### Virus isolation and identification

Liver and kidney samples from different commercial duck farms were collected and homogenized in sterile phosphate buffered saline (PBS, pH 7.2) to give a 20% suspension (w/v), and centrifuged at 8000 × *g* at 4°C for 10 min. Supernatants were filtered through a 0.22-μm syringe-driven filter. The virus filtrate was inoculated into the chicken liver cell line (LMH, ATCC), After a 1-h adsorption at 37°C, Dulbecco’s modified eagle medium (DMEM; Hyclone, Beijing, China) containing 2% FBS was added. The cultures were incubated at 37°C with 5% CO_2_. To detect novel GoAstV, RT–PCR was performed to amplify a partial GoAstV genomic sequence using a pair of specific primers based on the ORF2 gene (Table 2).

**Table 2.** Primers used in this study for detection of novel GoAstV.

Primer name	Sequence (5′→3′)	Target	Product size(bp)	Purpose
GoAStV-F1	ATTCTTGGCTCGGTTGTC	ORF2 gene of Novel GoAstV	489bp	RT-PCR
GoAStV-R1	CCTGTGTTGCTCCTTCTC			

### Astrovirus genome sequencing

The complete genome sequence of the isolate (A in Taian County) was investigated by next-generation sequencing (NGS) with the Hiseq platform following the manufacturer’s instructions. The NGS raw sequence reads were analysed, and de novo was assembled with different modules of CLC Genomics Workbench software v 9.5.2 (Qiagen, Boston, MA, USA). All large contigs (>5000 bp) were identified and extracted after the submission in BLAST was conducted at NCBI module of the software. Given the BLASTN searching result, the reference avian AstV strains were taken to build the full-length genome of AstV. The final assembly of AstV genome were ordered and oriented manually. The NGS was performed at the Shanghai Personal Biotechnology Co., Ltd, China. Eight pairs of primer were developed to verify the NGS results for the obtained whole sequences [15] (Table 3). The complete ORF2 gene of novel GoAstV (i.e. B in Liaocheng County, C in Xuzhou County, and D in Zhengzhou County) was amplified with the above primers to investigating the variability of this virus.

**Table 3.** Primers used for RT-PCR amplification and sequencing.

Primer name	Sequence (5′→3′)	Target	Product size (bp)
GoAstV F1	TGTAGCCTCTGTGTTTTCGCTTCCTGC	1–27	157
GoAstV R1	TCAAGTGAGGAAAAGGGGTTGACAATGGG	129–157	
GoAstV F2	CACTCGTGAGGAAAGGAAGACTGATG	158–183	197
GoAstV R2	CATACCTCCCATTGTCAACCCCTATAGC	327–354	
GoAstV F3	AATGCCATCTATCTTAAAGATTGG	297–320	1472
GoAstV R3	CCTTCAACAACGACAATGG	1750–1768	
GoAstV F4	CAGTCCCTGTACAGATTTTA	1531–1550	1366
GoAstV R4	TCAACTTGTTCATCCTTTAC	2877–2896	
GoAstV F5	AGATTGATGAAGCCATTGAG	2689–2708	1355
GoAstV R5	CAGCCCGCCGTTCTGTCTGT	4024–4043	
GoAstV F6	AGGCTGTATCAGATATTGAT	3840–3859	1278
GoAstV R6	TCATTTTGTCATTAACGGG	5099–5117	
GoAstV F7	GGGCGGTGGCCCCGCGCG	4920–4937	1369
GoAstV R7	CTTGACCTGGATTCTGCC	6271–6288	
GoAstV F8	ACAACTGGACAAGGTACC	6113–6130	1121
GoAstV R8	TTTAAAGATTTTAAATGCTT	7214–7233	

### Phylogenetic analysis

The present study compared nucleotide and deduced amino acid sequences of the ORFs with other known AstV ORF sequences that were retrieved from the GenBank database. The nucleotide and deduced amino acid sequences were performed using ClustalW in the Megalin module within the Lasergene 7.0 software (DNASTAR Inc., Madison WI, USA). Phylogenetic trees for the deduced amino acid sequences of the three ORFs were constructed using the neighbour-joining method in MEGA version 7.0 program, with bootstrap values calculated from 1000 replicates.

### Experimental infection study

One-day-old goslings and One-day-old ducklings were obtained from a local hatchery (Shandong, China), and PCR/RT–PCR detected free of GoAstV-specific nucleic and other waterfowl-origin viruses. Birds were raised in negative pressured isolators. Two experiments were conducted to determine the pathogenicity of the isolate. Clinical signs and mortality were recorded for 15 days. Tissue samples (e.g. liver and kidney) of dead birds and survival birds after 15 days were immediately frozen for pathological examination and virus identification. All the animal infection experiments were approved by the Animal Care and Use Committee of Shandong Agricultural University and conducted in accordance with the “Guidelines for Experimental Animals” of the Ministry of Science and Technology (Beijing, China).

### Duckling experiment

In order to determine the pathogenicity of the isolated virus, twenty 1-day-old healthy ducklings were infected by being subcutaneously inoculated with 0.3 ml of the isolated virus (AstV-SDTA strain; 5 × 10^4.3^ TCID_50_/ml) as the experimental group, and twenty 1-day-old ducklings were inoculated with sterile PBS in the same manner as the control group.

### Gosling experiment

This study was designed to verify whether the isolate is pathogenic in goslings. Twenty 1-day-old healthy goslings were infected as in duckling experiment, and twenty 1-day-old goslings inoculated with sterile PBS were used as the uninfected control.

### Histopathology

Fresh tissues collected from the diseased or experimental ducklings were fixed with a10% neutral buffered formalin. Then the tissue was cut into 4 μm thick with paraffin and stained with haematoxylin and eosin (H&E) using standard methods. Finally, the histopathological change of organs (e.g. liver and kidney) was observed under an optical microscope (Nikon, EclipseE100, Japan).

## Results

### Clinical and pathological investigations

**Figure 1.**
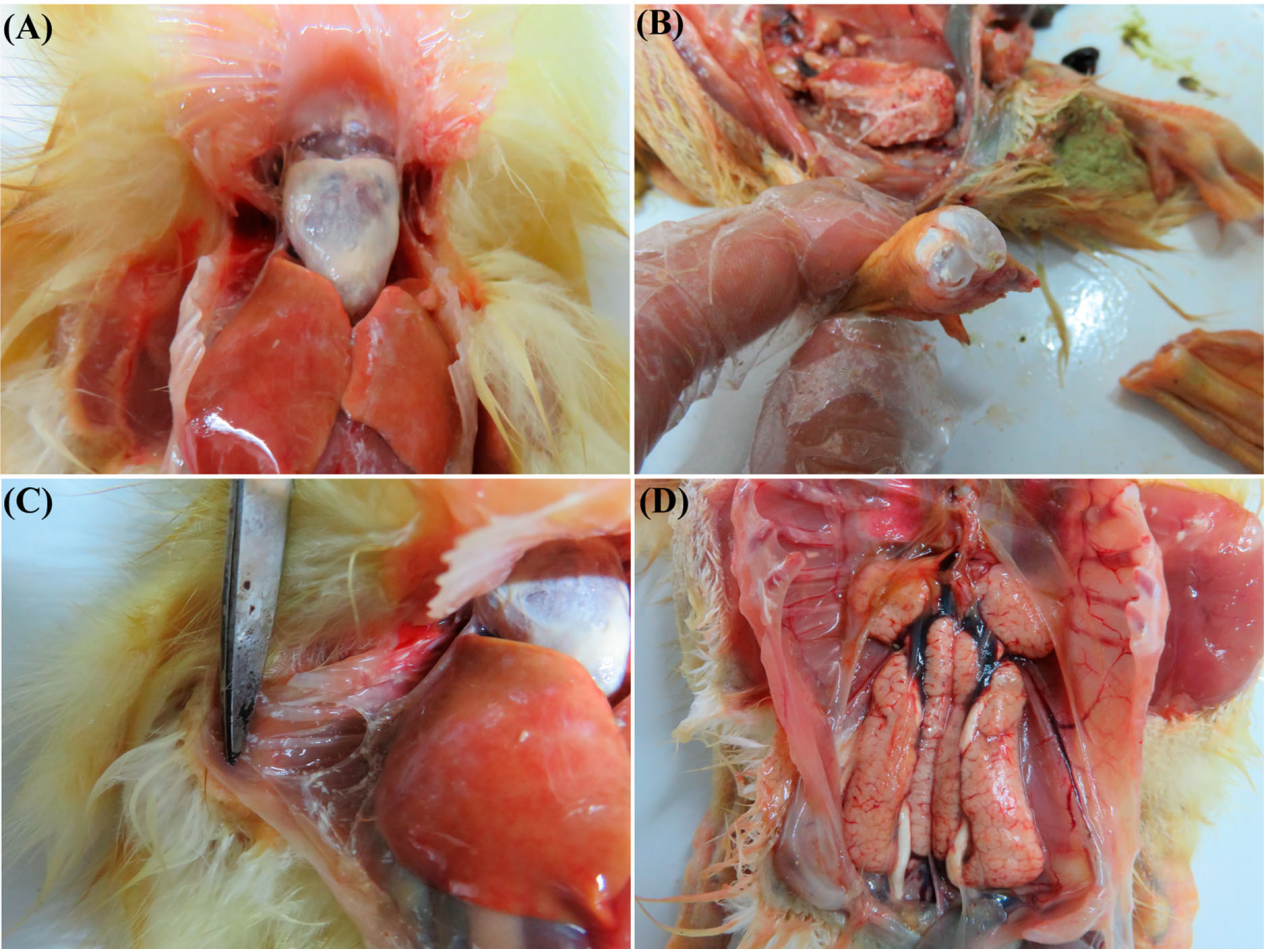
Postmortem lesions of ducklings infected clinical samples. A, Urate deposits in heart. B, Urate deposits in articular cavity. C, Urate deposits in thoracic mucosa. D, Severe haemorrhage and swellings of kidneys and urate deposits in ureters.

In the field cases, the clinical signs of the disease were characterized by signs of depression, loss of appetite, white faeces, and death occurred from when the ducklings were aged 2–4 days old, and peaked at the age of 8–11 days old with the mortality rates from 5% to 20%. The highest mortality rate in large group can exceed 30%. The survival ducklings grew slowly and exhibited susceptibility to bacterial infection. At necropsy, a few affected ducklings showed typical gout symptoms, with urate deposits in the heart, ureters, joints and other internal organs (Figure 1(A–C)). The majority of the dead ducklings showed severe haemorrhage and swellings of kidneys (Figure 1(D)).

### Bacterial culture and viral nucleic acids detection

**Figure 2.**
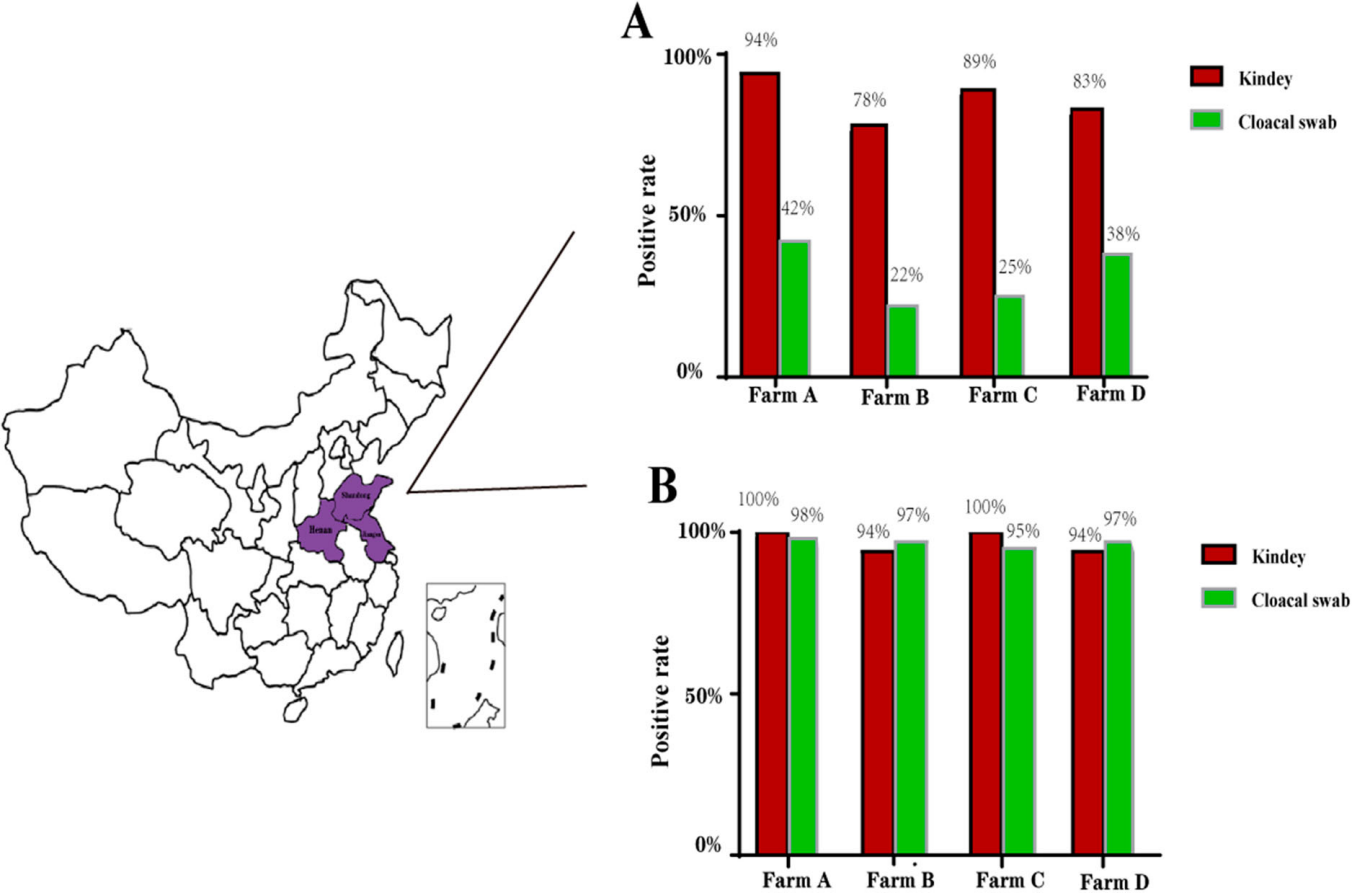
Duck-origin goose astrovirus distribution in China and the infection rates of novel GoAstV at various farms. A, Tested by RT-PCR. B, Tested by qRT-PCR.

Virulent bacteria were not isolated and the tissue samples determined by PCR/RT–PCR for fowl adenovirus, duck plague virus, duck Tembusu virus, duck astrovirus, duck reovirus, duck circovirus, goose haemorrhagic poly-omavirus and goose parvovirus all showed negative results. It is noteworthy that novel GoAstV is positive, 62 out of 72 (86.1%) kidney samples and 76 out of 240 (31.7%) cloacal swabs were identified to be novel GoAstV-positive by RT–PCR, and 302 samples (i.e. 70 kidneys and 232 cloacal swabs) detected as novel GoAstV-positive by qRT-PCR with a positive rate of 96.8% (302/312) (Figure 2).

### Isolation of duck-origin goose astrovirus in LMH cells

After five passages, 9 out of 12 samples were novel GoAstV positive with the above primers. The results of further sequencing confirmed that the virus is novel GoAstV. All these clearly demonstrated that the virus has been efficiently isolated from the diseased ducklings using LMH cell.

### NGS analysis

A total of 2,731,347 sequencing reads of 151-mer were generated by the HiSeq sequencer. The sequence data from the viral stocks output file in fastq format was 1.28 Gb in size. Low-quality reads, trim poly-T tails and adapter sequences were processed by quality control (QC) filters of the Hiseq platform for removal, following alignment to duck mRNA or rRNA sequences. The residual 271,275 reads (9.75%) were aligned with sequences in the viral genome database, and the mapped reads were further assembled by de novo assembly module of CLC Genomics Workbench.

### Genome sequence analysis

As revealed from the sequencing results, the full-length genome of AstV-SDTA achieved a length of 7227 nucleotide (nt), excluding the poly(A) tail, and was submitted to the GenBank (MN809622). There are three coding regions ORF 1a, ORF 1b, and ORF 2, and the termini of the genome sequence were 5′UTR (245 nt), 3′UTR (228 nt), and a poly (A) tail. ORF 1a of the isolate was 3255 nt long and was predicted to encode a polypeptide of 1084 aa. ORF 1b of the isolate was 1551nt long, encoding an RdRp of 516 aa. As expected, seven highly conserved sequence (5′-AAAAAAC-3′) from nt 3341–3347 as a potential ribosomal frameshift signal were observed between ORF 1a and ORF 1b overlapping region, and a downstream stem-loop structure (3355–3380 nt), which was predicted by RNA folding analysis. The start codon of ORF2 was 18 nt downstream of the stop codon of ORF1b and encoded a 704 aa capsid protein. Moreover, complete ORF2 sequences of the three strains (i.e. B in Liaocheng County, C in Xuzhou County, and D in Zhengzhou County) were 2115 nt in length and respectively classified as AstV-SDLC (GenBank accession no. MT323162), AstV-JSXZ (GenBank accession no. MT323161), and AstV-HNZZ (GenBank accession no. MT323163) strain.

### Phylogenetic analysis

**Figure 3.**
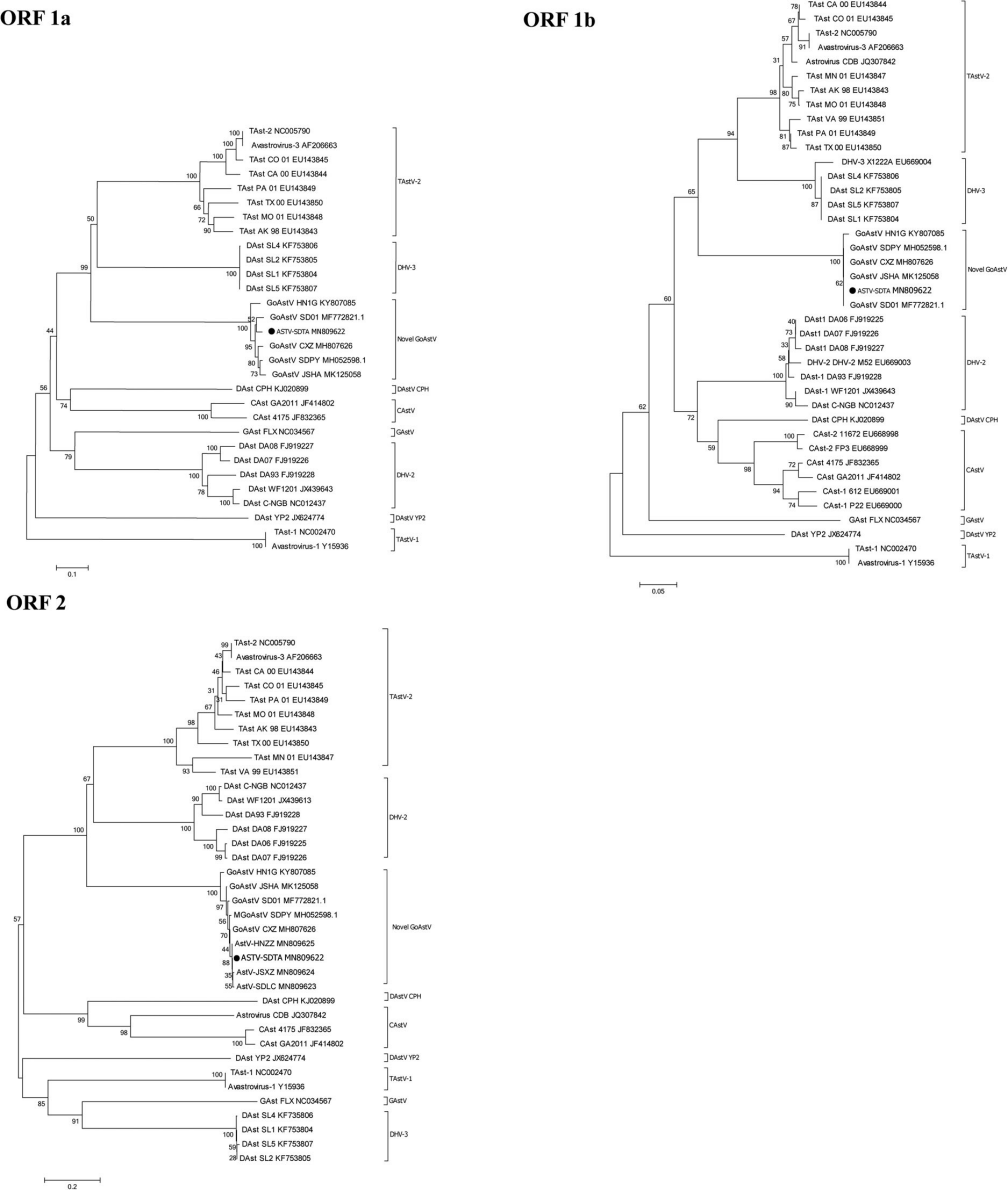
Phylogenetic relationship analysis based on the amino acid sequences of ORF1a, ORF1b and ORF2 of the ASTV-SDTA strain (●) and other AstVs. The trees were generated using MEGA 7.0 software and the Neighbour-joining method with 1000 bootstrap replicates. The ASTV-SDTA isolate determined in this work is indicated by a black dot.

By the neighbour-joining phylogenetic analysis, the phylogenetic trees of the three viral ORFs were confirmed. As shown in Figure 3, the AstV-SDTA strain located on the identical branch as the other novel GoAstV, and the AstV-SDTA strain was clustered with TAstV-2 and DHV-3 AstV strains in ORF 1a and ORF 1b genes. While, amino acid phylogenetic analysis of ORF 2 showed that the AstV-SDTA formed a sister clade neighbouring with TAstV-2 and DHV-2 AstV strains. The amino acid of ORF 1a, ORF 1b, and ORF 2 of AstV-SDTA with 99.1–99.8%, 96.5–99.5%, and 96.6–99.0% homologies to those regions of other novel GoAstV strains, and the amino acid of capsid protein of the AstV-SDTA strain shared identities of 55.7–55.9% and 56.2% with DHV-2 and TAstV-2 strains, respectively (Table 4). The homologies of nucleotide and amino acid sequences of four strains were compared those of other known avian AstVs as determined by BLAST analysis were summarized in Table 4. In summary, the results revealed that the AstV-SDTA strain shares a tight association with other novel GoAstV regardless of any single sequence or whole genome.

**Table 4.** Sequence identities between duck-origin goose astrovirus AStV-SDTA strain with selected representative.

	AstV-SDTA						
	ORF 1a		ORF 1b		ORF 2		Complete sequence
Strain	nt	aa	nt	aa	nt	aa	nt
AstV-SDLC	**–**	**–**	**–**	**–**	99.7	99.6	**–**
AstV**-**JSXZ	**–**	**–**	**–**	**–**	99.5	99.3	**–**
AstV-HNZZ	**–**	**–**	**–**	**–**	99.7	99.4	**–**
GAstV FLX	54.3	47.5	64.3	64.3	49.3	40.4	54.2
TAstV CO/01	61.2	59.3	69.2	72.4	58.9	56.2	61.7
TAstV PA/01	61.3	59.6	68.8	72.8	58.6	56.2	61.6
TAstV-1	50.9	40.8	60.1	57.6	47.7	38.5	52.1
DAstV DA08	54.9	47.6	65.9	67.9	60.3	55.9	58.3
DAstV DA93	55.4	47.8	64.9	68.4	59.9	55.7	58.2
DAstV WF1201	55.2	47.9	65.3	67.7	60.7	55.7	58.4
DAstV SL5	62.7	58.3	68.8	71.7	50.2	37.4	59.1
CAstV 4175	56.9	48.1	65.9	60.0	45.7	29.5	54.6
CAstV GA2011	56.8	48.4	65.5	68.4	47.7	35.3	55.2
GoAstV SDPY	98.6	99.3	99.8	97.9	99.1	97.9	98.2
GoAstV SD01	98.5	99.8	99.2	96.5	98.5	96.6	98.3
GoAstV HING	97.2	99.8	99.1	99.1	97.2	97.5	97.2
GoAstV CXZ	98.3	99.2	99.5	99.5	99.2	99.0	98.8
GoAstV JSHA	98.1	99.1	99.5	99.5	98.7	98.9	98.2

Note: aa: amino acid sequence; nt: nucleotide sequence.

### Outcome of infection experiments with AstV-SDTA

#### Duckling infection experiment

**Figure 4.**
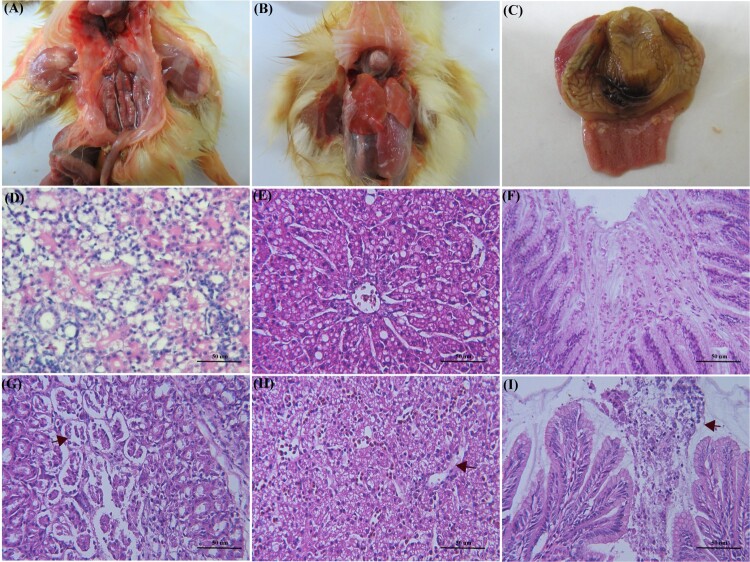
Histopathological changes in experimentally infected ducklings with AstV-SDTA strain. A, Severe haemorrhage and swellings of kidneys and urate deposits in ureters. B, Urate deposits in visceral organs. C, Urate deposits in proventriculus**.** G, collapse of renal tubular epithelial cells and swelling of glomeruli. H, Vacuolar degeneration of hepatocytes. I, Severe mucosal necrosis of proventriculus. (D–F) Negative control (histology changes in healthy duck).

Twelve out of the 20 infected ducklings exhibited signs of depression and started to excrete white faeces at 2 days post infection (dpi). Besides, one death occurred at 4, and 7 dpi, respectively, resulting in a mortality rate of 10% (2/20). At the end of the experiment, the weight gain of the infected ducklings was lower than that of the uninfected group. At necropsy, a small amount of urate deposits was observed on the surface of the heart and proventriculus, and severe haemorrhage and swellings of the kidneys were observable in a dead duckling at 4 days old (Figure 4(A–C)), and the other infected ducklings only showed haemorrhage and swellings of the kidneys without developing significant symptoms of gout. The results of histologic examination suggested that the renal epithelial cells were degenerated and necrosed, as well as glomerular swelling in kidney (Figure 4(G)). The liver showed vacuolar degeneration of hepatocytes (Figure 4(H)). As showed in Figure 4(I), severe mucosal necrosis could be observed clearly in the proventriculus. As indicated from the mentioned results, AstV-SDTA was successfully recovered from infected ducklings and confirmed by RT–PCR and sequencing.

#### Gosling infection experiment

Depression and lethargy occurred in the infected group on the 2nd day, one gosling died on the 6th and 11th day after the infection, and two goslings died at 8 dpi; as a result, a mortality rate of 20% was achieved (4/20). The major feature of the lesion was urate deposition (e.g. liver, heart and kidney). The symptoms were similar with those previously cases of novel GoAstV infection.

## Discussion

The sudden outbreak and quick spread of the novel GoAstV, primarily causing gosling gout, caused huge economic losses to the Chinese goose industry since 2016 [13]. Generally, AstVs infections have been found to be associated with duck hepatitis [24,25]. Thus far, increasing reported evidences have indicated that the potential cross-species transmission of AstV between domestic fowl is possible [11, 26]. In March 2019, a disease characterized by visceral gout was reported in a commercial duck farm in midland of Shandong Province, and the causative agent of the disease was confirmed to be caused by a novel GoAstV strain. The amino acid sequence of the capsid protein of the isolate strain shared high identities of 99.6% with other novel GoAstV [27]. In the present study, a disease characterized by haemorrhage and swellings of kidneys was reported in the course of an epidemiological investigation. Pathogen isolation, pathogenic differential diagnosis, disease reproduction, and phylogenetic analysis were performed on diseased duck flocks. Lastly, the results of the present studies strongly supported a novel GoAstV as the causative agents of the duckling disease prevalent during this outbreak in China.

In the present study, the majority of the dead ducklings in clinical cases did not develop significant gout symptoms, and they were primarily characterized by nephritis, which were not consistent with existing reports. Moreover, samples from the healthy ducklings in the infected flocks were found that the positive rate of novel GoAstV was over 96% with qRT-PCR assay. The mentioned phenomena revealed that the novel GoAstV strain had a powerful capacity for horizontal transmission. Of course, the higher viral infection rate in the duck flocks may result from the ability of the virus to have vertical transmission as well. Subsequent researches are required to determine its host pathogenicity and route of transmission.

The major mutation region of AstV-SDTA isolate was concentrated in the ORF2 region, which encoded capsid protein. The deduced amino acid sequence of the capsid protein of the AstV-SDTA isolate shared high identities of 96.6–99.0% with other novel GoAstV strains, and the nucleotide homology analysis of complete genome sequence of AstV-SDTA isolate shared the highest identities of 97.2–98.8% with other novel GoAstV strains. The mentioned findings suggested that the cross-species transmission of the novel GoAstV strain from goslings to ducklings is likely to achieve, and the virus has progressively adapted to the host and remains pathogenicity to the gosling. Such host variant might be the result of mutation and evolution from novel GoAstV strains of different origins, which then gained the ability to infect and replicate in ducklings. Further experiments are required to test this prediction.
